# Editorial: Metabolic Regulation in the Development of Cardiovascular Diseases

**DOI:** 10.3389/fcell.2021.768689

**Published:** 2021-10-13

**Authors:** Yimei Ma, Md. Shenuarin Bhuiyan, InKyeom Kim, Xiaoqiang Tang

**Affiliations:** ^1^Key Laboratory of Birth Defects and Related Diseases of Women and Children of Ministry of Education, State Key Laboratory of Biotherapy, Department of Pediatrics, West China Second University Hospital, Sichuan University, Chengdu, China; ^2^Department of Pathology and Translational Pathobiology, Louisiana State University Health Sciences Center, Shreveport, LA, United States; ^3^Department of Pharmacology, Cardiovascular Research Institute, BK21 Plus KNU Biomedical Convergence Program, Department of Biomedical Science, School of Medicine, Kyungpook National University, Daegu, South Korea

**Keywords:** metabolism, cardiac disease, heart failure, post-translational modification, mitochondria

Metabolic syndromes increase the risk of cardiovascular diseases (CVDs) (North and Sinclair, 2012), and metabolic reprogramming can either reverse or rescue the molecular events that lead to CVDs (Chen et al., 2020a). However, the metabolic mechanisms underlying CVDs are not fully understood. We have prepared a special Research Topic. This Research Topic entitled “*Metabolic Regulation in the Development of Cardiovascular Diseases*” received 11 original articles, 7 review articles, and 2 opinion articles. This special issue highlights recent research findings to clarify the relationship between metabolism and CVD.

Metabolic dysregulation and metabolic syndromes are independent risk factors for CVDs (Zhou et al., 2018). Genetic mutations in metabolic enzymes, as well as transcription factors, can cause CVDs (Austin et al., 2019). In this issue, Zhang et al. identify the occurrence of mutations in transcription factor EB (TFEB), which controls lysosomal biogenesis and metabolism (Settembre et al., 2013), as a potential risk factor for acute myocardial infarction. This study detected novel variants of the metabolic regulator, TFEB, that might contribute to the development of acute myocardial infarction. Furthermore, pregnancy-related CVDs, such as arterial dissection, are also affected by metabolic conditions (Wang et al., 2021). Deng et al. emphasized the importance of glycemic control in pregnant women, which could improve the understanding, prevention, and treatment of pregnancy-related arterial dissection.

Genetic mutations, or dysregulation of metabolic enzymes and their regulators, directly alter cell metabolism, intracellular metabolites, and physiological functions of vascular cells such as endothelial cells (ECs) (Tang et al., 2014). Endothelial metabolic homeostasis and reprogramming can regulate endothelial functions, including angiogenesis, inflammation, and barrier maintenance (Tang et al., 2014; Subramanian et al., 2021). Peng et al. provided an overview of the metabolic pathways in ECs under normal and pathological conditions. Their review highlighted the metabolic reprogramming of endothelium as a potential therapeutic approach for controlling CVDs.

Glucose metabolism in ECs is critical for cardiovascular homeostasis and diseases (Dumas et al., 2021). Glucose catabolism is regulated by enzymes such as 6-phosphofructo-2-kinase/fructose-2,6-biphosphatase 3 (PFKFB3), whose dysfunction drives endothelial injury and vascular inflammation (Bartrons et al., 2018). The role of PFKFB3 in non-EC vascular cells remains unclear. In this regard, Poels et al. show that PFKFB3 expression in monocytes is positively correlated with the occurrence of coronary arteries with unstable plaque phenotypes. Inhibition of PFKFB3 reduced the number of late plaques in vulnerable phenotypes and resulted in stable plaque phenotypes. This phenomenon was coupled with a decrease in glycolytic flux in mononuclear cells within the circulating peripheral blood. The study by Tillie et al. also examined the role of myeloid PFKFB3 in atherosclerosis development. Partial knockout of *Pfkfb3* in myeloid cells did not affect the development of atherosclerosis. Thus, further studies are required to dissect the contribution of PFKFB3 in other non-EC vascular cells, such as vascular smooth muscle cells, fibroblasts, and other immune cells, in atherosclerosis.

In addition to metabolic enzymes, their products (metabolites) are critical for organ homeostasis and injury repair (Chen et al., 2021b; Dumas et al., 2021). Zhang et al. discussed the role of endothelium-derived lactate in regulating the metabolic microenvironment in tissue regeneration and CVD. Endothelium-lactate interactions affect the microenvironment *via* multiple routes. Lactate can be produced by ECs during glycolysis and exported *via* its transporters to the extracellular milieu to regulate cells in the microenvironment. Furthermore, lactate can be imported from extracellular blood compartments by ECs to regulate angiogenesis, or transferred transendothelially to the microenvironment to regulate stromal and immune cells. These interactions regulate the cell microenvironment, tissue regeneration, and CVDs. The biochemical mechanisms underlying lactate function remain unknown, although some findings revealed the potential involvement of histone lactylation (Eming et al., 2021).

In addition to pyruvate and lactate, other metabolites also contribute to cardiovascular homeostasis (Li et al., 2019). One such metabolite is epoxyeicosatrienoic acids (EETs), a derivative of arachidonic acid synthesized by cytochrome P450 (Duflot et al., 2014). EETs are rapidly hydrolyzed into less bioactive dihydroxyeicosatrienoic acid (DHET) by soluble epoxide hydrolase (sEH) (Wang et al., 2013). Hamzaoui et al. show that inhibition of sEH inhibits cardiac remodeling, as well as diastolic and systolic dysfunction associated with chronic kidney disease (CKD). Therefore, inhibition of sEH has therapeutic potential for preventing cardiorenal syndrome, which may be regulated by intracellular DHET. Furthermore, metabolites from the gut microbiota are critical regulators of mammalian metabolism and CVDs (Tang et al., 2019). Trimethylamine *N*-oxide (TMAO) is an intestinal microbiome-derived metabolite synthesized from specific food components, such as red meat (Tang et al., 2019). It acts as a risk factor for vascular diseases, such as atherosclerosis (Tang et al., 2019). However, the role of TMAO in cardiac diseases remains unclear. Videja et al. demonstrated that high TMAO levels preserve the production of mitochondrial energy and cardiac function in an experimental model of right ventricular heart failure, thereby suggesting that TMAO promotes effects similar to metabolic preconditioning under specific conditions. This finding is opposite to all other studies published so far on TMAO in CVD (Witkowski et al., 2020).

Mitochondria are essential for maintaining normal cardiomyocyte homeostasis and ensuring healthy heart function (Bonora et al., 2019). Two review papers in this special issue discussed the recent advances regarding mitochondrial biology in CVDs. Xin et al. addressed the function of the mitochondrial fusion protein, mitofusin-2 (MFN2), in regulating mitochondrial morphology, metabolism, calcium homeostasis, and mitochondrial DNA stability in CVDs, and highlighted MFN2 as a therapeutic target for treating CVDs. In addition, Liao et al. discussed the pathways that regulate mitochondrial function in response to mechanical stress during the development of cardiomyopathy and heart failure. One of the central regulators is the Hippo pathway, which plays a pivotal role in heart failure (Wang et al., 2018). The Hippo pathway targets not only mitochondria but also other organelles and pathways (Zhao et al., 2021). Mammalian sterile 20-like kinase 1 (MST1) is a crucial component in the Hippo pathway (Zhao et al., 2021). Feng et al. observed that knockout of *Mst1* inhibited mitochondrial division and reduced left ventricular remodeling in diabetic cardiomyopathy, thereby highlighting the critical role of the Hippo pathway in the regulation of mitochondria and CVDs.

Dysregulation of metabolism can lead to systemic and vascular inflammation, and *vice versa* (Tang et al., 2014). Aging-related chronic inflammation is a hallmark of chronic metabolic disorders, including obesity and type 2 diabetes, contributing to CVDs (Nafisa et al., 2018). Immune cells, especially T cells, accumulate in adipose tissue during aging (Villarroya et al., 2018), and pre-adipose T cells promote aging-related remodeling of adipose tissues (Chen et al., 2021a). Pan et al. compared the differences in adipose tissue morphology and function between young and aged mice, and reported the “whitening” effect of brown adipose tissue (BAT) in old mice. The proportion of T cells in the BAT of old mice was higher, with more aging markers than in those of young mice. Senescent T cells release high levels of interferon-gamma, which inhibits preadipocyte-to-brown adipocyte differentiation. Because BAT is a beneficial factor against CVD (Ruan et al., 2018), further studies are needed to test how T cells in BAT participate in CVD, including cardiac remodeling.

Wu et al. discussed the recent advances regarding the role of CC chemokine receptor-9 (CCR9)/CC motif chemokine 25 (CCL25) in inflammation and CVD. Targeting the CCR9/CCL25 axis pharmacologically can decrease or modulate inflammation. High serum phosphate concentrations are associated with cardiovascular risk in both the general population and patients with CKD (Vervloet et al., 2017). Another review paper, contributed by Zhou et al., discussed the advances in the understanding of phosphate homeostasis in healthy and CKD conditions. The authors highlighted that fibroblast growth factor 23 plays an important role in controlling serum phosphate levels to mitigate phosphate-induced CVD.

The upregulation of endothelin 1 (ET1), a vasoconstrictor factor, contributes to hypertension and organ fibrosis (Tang et al., 2020); however, the mechanism underlying ET1 regulation remains unclear. Xu et al. reported that glycogen synthase kinase 3 (GSK3) and serum response factor (SRF) contributed to angiotensin II-induced ET1 overexpression in ECs. This study highlighted a previously unrecognized mechanism that contributes to the transcriptional regulation of endothelin and could lead to new approaches for CVD interventions targeting the GSK3-SRF axis.

In addition to local inflammation, systemic and local accessibility and bioavailability of gas molecules also regulate cell metabolism (Das et al., 2018). For instance, chronic hypoxia is an essential factor in many CVDs. The main energy fuels of heart are fatty acids. However, under chronic hypoxia, glucose oxidation is downregulated and glycolysis is upregulated (Tang et al., 2017). The mechanisms underlying adaptive cardiac metabolism remain unclear. Su et al. provided a thorough discussion on this topic. They concluded that the heart initiated transcriptional programs to increase the utilization of carbohydrates, rather than fatty acids, to produce ATP. This involves altered mitochondrial structure and function, improved metabolic efficiency, and reduced reactive oxygen species production in hypoxic cardiac tissues. The core participants in hypoxia are hypoxia inducible factors (HIFs) and their modulators, including the HIF-prolyl hydroxylase (PHD) isotypes PDH1, PDH2, and PDH3 (DeBerge et al., 2021). PHD pan-inhibitors have been used to treat anemia in patients with CKD. Similarly, systemic *Phd1* or *Phd2* knockout was found to improve atherosclerosis (Marsch et al., 2016; Rahtu-Korpela et al., 2016). In this issue, Demandt et al. analyzed the roles of PHD3 in hypercholesterolemia using low-density lipoprotein receptor (*Ldlr*) and *Phd3* double knockout mice. The authors found that systemic *Phd3*-deficiency induced adverse lipid profiles and increased hepatocellular volume without altering the development of atherosclerotic plaques, compared to other PHD isotypes. Interestingly, the effect of PHD3 on hypercholesterolemia is opposite to that of PHD1 and 2 (Marsch et al., 2016; Rahtu-Korpela et al., 2016), and to the detrimental effect of PDH3 overexpression on the progression of atherosclerosis in *ApoE*^−/−^ mice (Liu et al., 2016). Further studies are needed to systematically investigate the multi-dimensional roles of PHDs in hypercholesterolemia and related CVDs such as atherosclerosis.

In addition to HIFs and PHDs, there are other types of hypoxia regulators, such as hypoxia-induced mitogenic factor (HIMF), a member of the resistin-like molecule protein family expressed in mammals (Lin and Johns, 2020). HIMF is involved in numerous physiological processes including mitosis, angiogenesis, inflammation, and vasoconstriction (Lin and Johns, 2020). In addition, HIMF responds to several pathological conditions involving the lungs and the cardiovascular system. In this issue, Lv and Liu discuss the molecular characteristics and pathophysiological effects of HIMF, and highlight the potential clinical implications in CVDs and other diseases. Taken together, there has been remarkable progress in understanding the biology of hypoxia in CVDs. However, currently available drug strategies are still limited and have not been tested in patients with chronic hypoxia-related CVDs.

Considering that CVDs are critically controlled by metabolism and their regulators, metabolism-targeted drugs/interventions are promising strategies for the treatment of CVDs (Tang et al., 2017; Chen et al., 2020b). One drug with such potential is metformin (Kulkarni et al., 2020), a clinical anti-diabetic drug that targets mitochondria and regulates cell metabolism (Foretz et al., 2014). Metformin has the potential to repress cardiovascular aging and diseases (Nafisa et al., 2018); however, the sex-related effects of metformin remain unknown. Zhu et al. investigated the protective effects of metformin on cardiac metabolism and longevity in female mice; however, they found that metformin did not improve cardiac function or longevity in elderly female mice. Although multiple beneficial effects of metformin have been reported in age-related diseases, further systematic evaluation of the sex-related roles of metformin in heart conditions and longevity of older patients should be considered. Additional study of the synergistic effects of metformin with other drugs would also be interesting. Jia et al. investigated the synergistic effects of metformin and atorvastatin on diabetic cardiomyopathy and found the combination to provide better protection against diabetic cardiomyopathy than monotherapy, indicating that drug combinations may achieve greater clinical benefits than the use of a single drug.

In conclusion, the papers published on this Research Topic can potentially improve our understanding of genetic risk factors, metabolic enzymes and metabolites, metabolic inflammation, mitochondrial dynamics, the biology of hypoxia in CVD, and the treatment of cardiovascular diseases.

## Author Contributions

All authors listed have made a substantial, direct and intellectual contribution to the work, and approved it for publication.

## Funding

This work was supported by the National Natural Science Foundation of China (grant numbers 81970426 and 81800273); the Young Elite Scientists Sponsorship Program of China Association for Science and Technology (grant number 2018QNRC001); the Scientific and Technological Innovation Talents Program of Sichuan Province (grant number 2020JDRC0017); National Institutes of Health (NIH) grants R01HL145753, R01HL145753-01S1, and R01HL145753-03S1; LSUHSC-S CCDS Finish Line Award, COVID-19 Research Award, and LARC Research Award to MSB; the Basic Science Research Program through the National Research Foundation of Korea (NRF), funded by the Ministry of Education, Science and Technology (NRF-2021R1A2B502001763 and 2021R1A4A1021617); and the Korea Health Technology R&D Project through the Korea Health Industry Development Institute (KHIDI), funded by the Ministry of Health & Welfare, Republic of Korea (HI15C0001). This work was also supported under the framework of international cooperation program managed by the National Research Foundation of Korea (2021K2A9A2A07000135).

## Conflict of Interest

The authors declare that the research was conducted in the absence of any commercial or financial relationships that could be construed as a potential conflict of interest.

## Publisher's Note

All claims expressed in this article are solely those of the authors and do not necessarily represent those of their affiliated organizations, or those of the publisher, the editors and the reviewers. Any product that may be evaluated in this article, or claim that may be made by its manufacturer, is not guaranteed or endorsed by the publisher.
